# Gastric Subserous Vaccination With *Helicobacter pylori* Vaccine: An Attempt to Establish Tissue-Resident CD4+ Memory T Cells and Induce Prolonged Protection

**DOI:** 10.3389/fimmu.2019.01115

**Published:** 2019-05-17

**Authors:** Wei Liu, Zhiqin Zeng, Shuanghui Luo, Chupeng Hu, Ningyin Xu, An Huang, Lufeng Zheng, Eric J. Sundberg, Tao Xi, Yingying Xing

**Affiliations:** ^1^School of Life Science and Technology, China Pharmaceutical University, Nanjing, China; ^2^Jiangsu Key Laboratory of Carcinogenesis and Intervention, China Pharmaceutical University, Nanjing, China; ^3^Institute of Human Virology, Department of Medicine, University of Maryland School of Medicine, Baltimore, MD, United States; ^4^Department of Microbiology and Immunology, University of Maryland School of Medicine, Baltimore, MD, United States

**Keywords:** tissue-resident memory T cells, CD4+ T cells, subunit vaccine, *Helicobacter pylori*, immunological memory

## Abstract

Tissue-resident memory T (Trm) cells are enriched at the sites of previous infection and required for enhanced protective immunity. However, the emergence of Trm cells and their roles in providing protection are unclear in the field of *Helicobacter pylori* (*H. pylori*) vaccinology. Here, our results suggest that conventional vaccine strategies are unable to establish a measurable antigen (Ag)-specific memory cell pool in stomach; in comparison, gastric subserous injection of mice with micro-dose of Alum-based *H. pylori* vaccine can induce a pool of local CD4+ Trm cells. Regional recruitment of Ag-specific CD4+ T cells depends on the engagement of Ag and adjuvant-induced inflammation. Prior subcutaneous vaccination enhanced this recruitment. A stable pool of Ag-specific CD4+ T cells can be detected for 240 days. Two weeks of FTY720 administration in immune mice suggests that these cells do not experience the recirculation. Immunohistochemistry results show that close to the vaccination site, abundant CD4+T cells locate on epithelial niches, independent of lymphocyte cluster. Paradigmatically, Ag-specific CD4+ T cells with a phenotype of CD69+CD103- are preferential on lymphocytes isolated from epithelium. Upon *Helicobacter* infection, CD4+ Trm cells orchestrate a swift recall response with the recruitment of circulating antigen-specific Th1/Th17 cells to trigger a tissue-wide pathogen clearance. This study investigates the vaccine-induced gastric CD4+ Trm cells in a mice model, and highlights the need for designing a vaccine strategy against *H. pylori* by establishing the protective CD4+ Trm cells.

## Introduction

In addition to effector memory T (Tem) cells and central memory T (Tcm) cells, tissue-resident memory T (Trm) cells are the third subset of memory T cells that reside in the non-lymphoid tissues without entering recirculation (1). Proximity to the entry points of pathogens and their state of differentiation ensure that Trm cells can rapidly react to local infection (2–5). A study reveals that a subset of effector T cells within non-inflamed tissues manipulate the potential to differentiate into Trm cells after adaption to local survival cues (6). However, in most scenarios of immunization/infection, Trm cells emerged after the resolution of local inflammation (1, 7). Trm cell populations are well-characterized in terms of Trm cells derived from CD8+ T cells or generated in response to invasive pathogens, but are less well-understood in terms of Trm cells derived from CD4+ T cells or generated in response to non-invasive pathogenic bacteria (3).

*Helicobacter pylori* (*H. pylori*) is a highly successful pathogen that colonizes the stomach of humans (8). Development of vaccines is one of the desirable alternative strategies to eliminate the threat of *H. pylori*. Previous clinical trials have demonstrated that many attempts fail to provide sufficient protection against *H. pylori* in human (9, 10). Evidence obtained from mice suggests a strong ability of this bacterium to alter the detection of pattern recognition receptors (PRRs) and subvert host immune system by producing multiple virulence factors (11). When facing this pathogen, host immune system is unable to orchestrate a potent response to purge the infection. Most infected individuals develop asymptomatic chronic gastritis, which sustains over their lifetimes if no antibiotic intervention. It is commonly accepted the need for CD4+ T cells, rather than CD8+ T cells or antibody-mediated responses, in providing protection (12, 13). Multiple studies using conventional vaccine strategies show that vaccination reduces *H. pylori* colonization in mice (13–18). Yet, the emergence of gastric Trm cells in these studies remains enigmatic. Dependence solely on recalling circulating memory T cells induced by conventional vaccination may result in a delay and “miss the boat” for optimal protection. Establishing a CD4+ Trm pool in stomach by vaccination and exploring the generation, maintenance, and behavior of these cells are attractive. However, the first-line challenges are how to send these pathogen-specific CD4+ T cells into the tissue “battlefield” and make sure that a CD4+ Trm pool can be detected. To address these gaps in the field, by using intracellular cytokine staining, we assessed the magnitude of antigen (Ag)-specific CD4+ cells after various vaccinations and found a measurable pool of Ag-specific CD4+ Trm cells in mice that vaccinated with micro-dose of Alum-based *H. pylori* vaccine in gastric subserosa layer (GSL). The characteristics and mechanism of protection against *H. pylori* were further investigated in these cells. This study proposes a notion that investigators should take into account a subset of Trm cells when planning an *H. pylori* vaccine strategy.

## Materials and Methods

### Vaccine Preparation

Purified CCF protein and GEM particles were prepared and stored according to previous protocols (19, 20). Briefly, the CCF protein was expressed by *Escherichia coli* Rosetta (DE3) cells with pET-28a-CCF. The protein was first purified by nickel affinity chromatography (GE Healthcare), followed by anion-exchange chromatography with DEAE Sepharose FF (Amersham Pharmacia Biotech AB, Sweden). The purity of CCF was confirmed by Coomassie blue staining. The GEM particles were prepared by *Lactococcus lactis* NZ9000 cells using a hot-acid water bath. Vaccine with Alum was prepared with an equal volume of CCF solution and Alum adjuvant. CpG ODN 1826 was obtained from Sangon Biotech Co., Led. (China, Shanghai) and dissolved in CCF solution before intranasal vaccination.

### Animals and Immunizations

Eight-week-old female C57BL/6J mice were obtained from the Comparative Medicine Center of Yangzhou University and bred at the China Pharmaceutical University Animal Experimental Center. All animal experiments were approved by the Animal Ethical and Experimental Committee of China Pharmaceutical University. The immunizations were performed according to the timetables in the figures and the doses of antigen and adjuvants are indicated in the figure captions or special region of the figure.

### Gastric Subserous Layer Vaccination

Mice were anesthetized with 15 mg/kg Xylazine and 100 mg/kg Ketamine, and placed on a body temperature heating pad. After shaving the right abdomen, a 1.5 cm incision was made above the stomach. After laparotomy, the stomach was localized, and 5 μl vaccine preparation (Volume, CCF solution: Alum = 1:1, containing ~7.5 μg CCF) was injected into the gastric subserous layer of the greater curvature using a Hamilton syringe with a 33 G needle. Then, suturing with PGA absorbable sutures was performed using uninterrupted sutures for the peritoneum and interrupted sutures for the skin incision (Shanghai Pudong Jinhuan Medical Products Co., Ltd.).

### Preparation of Single-Cell Suspensions From Gastric Tissue

Single-cell suspensions were prepared as a previous study with modifications (21). Briefly, the whole stomach was isolated, cut through the lesser curvature, and the contents were removed before being placed into 15 ml RPMI 1640 containing 10 mM HEPES, 10% FBS, 4 mM EDTA, and 0.5 mM dithiothreitol. Gastric epithelial lymphocytes were isolated by shaking at 250 rpm and 37°C for 30 min. Tissues were then minced and incubated with another 15 ml RPMI 1640 containing 10 mM HEPES, 10% FBS, 4 mM EDTA, and 0.5 mM dithiothreitol for 15 min to isolate the remaining lymphocytes. Supernatants were passed through a 70 μm cell strainer. After washing and centrifugation, cell pellets were resuspended in an appropriate medium for further analysis or culture.

### Preparation of Single-Cell Suspensions From Lymphoid Organs and Blood

The spleen and mesenteric lymph nodes were isolated and gently pushed through a 70 μm cell strainer. After extensive washing, cells from the lymph nodes were collected. The cells from the spleen and blood were suspended in 7 ml erythrocyte lysis buffer (Biolegend) and washed twice with 10 ml PBS containing 5% FBS. Cells were collected for FACS analysis or stimulated *in vitro*.

### Antigen-Specific CD4+ T Cell Analysis

Single-cell suspensions from the stomach were purified with 67/44% Percoll gradients. The cells at the interface were collected and washed with 7 ml RPMI 1640 containing 10% FBS. To detect Ag-specific CD4+ T cells, purified single-cell suspensions from the stomach, MLN, spleen or blood were stimulated with 1 × 106 naïve, CFSE-labeled splenocytes that were preloaded with CCF in RPMI 1640 containing 10% FBS and 5 μg/ml BFA for 12 h. After collection, cells were stained for intracellular cytokines.

### FACS Analysis

For IFN-γ and IL-17 intracellular cytokine staining, *in vitro* restimulated cells were first stained with anti-CD4 (GK1.5) and anti-CD90.2 (30-H12) antibodies, then fixed and permeabilized with Intracellular Staining Fixation/Permeabilization Wash Buffer (Biolegend, San Diego, CA) and stained intracellularly with anti-IFN-γ (XMG1.2) and anti-IL-17 (9B10) antibodies. For cell phenotype detection, single-cell suspensions were stained with the following antibodies: anti-CD3ε (145-2C11), anti-CD90.2 (30-H12), anti-CD45 (30-F11), anti-CD4 (GK1.5 or RM4-4), anti-CD11b (M1/70), anti-CD8α (53-6.7), anti-CD19 (6D5), anti-MHC class II (M5/114.15.2), anti-CD69 (H1.2F3), anti-CD25 (3C7), anti-CD44 (IM7), anti-CD103 (2E7), anti-TCRγδ (UC7-13D5), anti-Ly6C (HK1.4), anti-Gr-1 (RB6-8C5), anti-CD11c (N418), and anti-F4/80 (BM8) purchased from Biolegend or BD Pharmingen. Multiparameter analyses were performed on a BD FACS Aria II or a BD FACS Calibur flow cytometer.

### Immunofluorescent Histology

For gastric histology, the longitudinal specimens were fixed with 4% paraformaldehyde, embedded in paraffin, and stained with hematoxylin and eosin (HE). For CD4 immunofluorescent staining, 20 or 10 μm frozen sections were cut and dried at room temperature. After blocking, these sections were stained with an Alexa Fluor® 488-anti-CD4 (GK1.5, Biolegend) antibody and/or purified anti-CD11b (M1/70, Biolegend) or anti-CD8α (53–6.7, Biolegend) antibody followed by goat anti-rat IgG2a/IgG2b Alexa Fluor® 488/594 antibody (Biolegend). The slides were washed and counterstained with DAPI to visualize cell nuclei, and images were acquired with a Panoramic 250 Flash III Scanner (3D Histech). The number of CD4+ cells in each section was counted in a 0.5 μm × 0.5 μm area with highest CD4+ cell signaling.

### Quantitative RT–PCR

Gastric RNA extraction and reverse transcription were carried out as described previously (22). PCR amplification was performed with a conventional TaqMan method. TaqMan gene primers and probes were designed by Sangon Biotech Co., Led. (China, Shanghai) based on the following sequence numbers: CCL5, Mm01302427_m1; CXCL9, Mm00434946_m1; CXCL10, Mm00445235_m1; GADPH Mm99999915_g1.

### FTY720 Treatment

For FTY720 treatment, 1 mg/kg FTY720 was injected i.p. daily to block circulating memory T cell egress from the lymphoid nodes according to the design of the experiments.

### Neutralizing Antibody Experiments

Immune mice were i.p. injected with 100 μg anti-CD4 antibody (GK1.5, BioXcell), anti-RatIgG1, anti-IFN-γ (XMG1.2, BioXcell) and anti-IL-17A (17F3, BioXcell) antibody every 2 days to deplete CD4+ T cells, IFN-γ and IL-17A according to the design of experiments.

### *H. pylori* Challenge

*Helicobacter pylori* SS1 was cultured as previously described (22). Sixty days after the last vaccination, the mice were challenged with 1 × 10^9^ CFU *H. pylori* SS1 (determined by turbidimetry) by gavage in 200 μl of 0.2% sodium bicarbonate solution.

### Quantitative Culture of *H. pylori*

Quantitative culture of *H. pylori* was performed as previously described (22). Briefly, half of the stomach was homogenized in 500 μl Brain Heart Infusion (BHI) broth and plated at a series of dilutions on BHI plates. The bacterial colonization was calculated at the whole organ level.

### Statistics

GraphPad Prism 7.0 software was used for statistical analyses. The differences between the groups were assessed using the Kruskal–Wallis test or Mann–Whitney *U*-test. *P* < 0.05 was considered statistically significant.

## Results

### Ag-specific CD4+ Effector T Cells Are Present in Stomach After Conventional Vaccinations, but Fail to Give Rise to a Formidable CD4+ Memory T Cell Pool

An outstanding question in the field of *H. pylori* vaccinology is whether conventional vaccinations can induce an Ag-specific CD4+ cell population in the stomach. Here, we used a recombinant *H. pylori* subunit vaccine, CCF, as a model Ag. CCF was constructed by multi-epitopes from *H. pylori* urease, and self-adjuvant regions from *Salmonella typhimurium* phase I flagellin FliC and cholera toxin B (20). To detect Ag-specific CD4+ T cells in stomach, we isolated total purified leukocytes from whole stomach of immune mice and co-cultured these cells with 1 × 10^6^ Ag-preloaded, CFSE-labeled naive splenocytes for 12 h in the presence of Brefeldin A (BFA). Two crucial effector cytokines, IFN-γ and IL-17A, for anti-*H. pylori* immunity were used to identify Ag-specific T cells. In the preliminary data, we found that for conventional vaccinations, Ag-specific CD4+ cells that produced only IFN-γ or IL-17A were rare, but the combination of IFN-γ and IL-17A allowed for the detection of more Ag-specific CD4+ T cells in these groups (Supplementary Figure 1).

**Figure 1:**
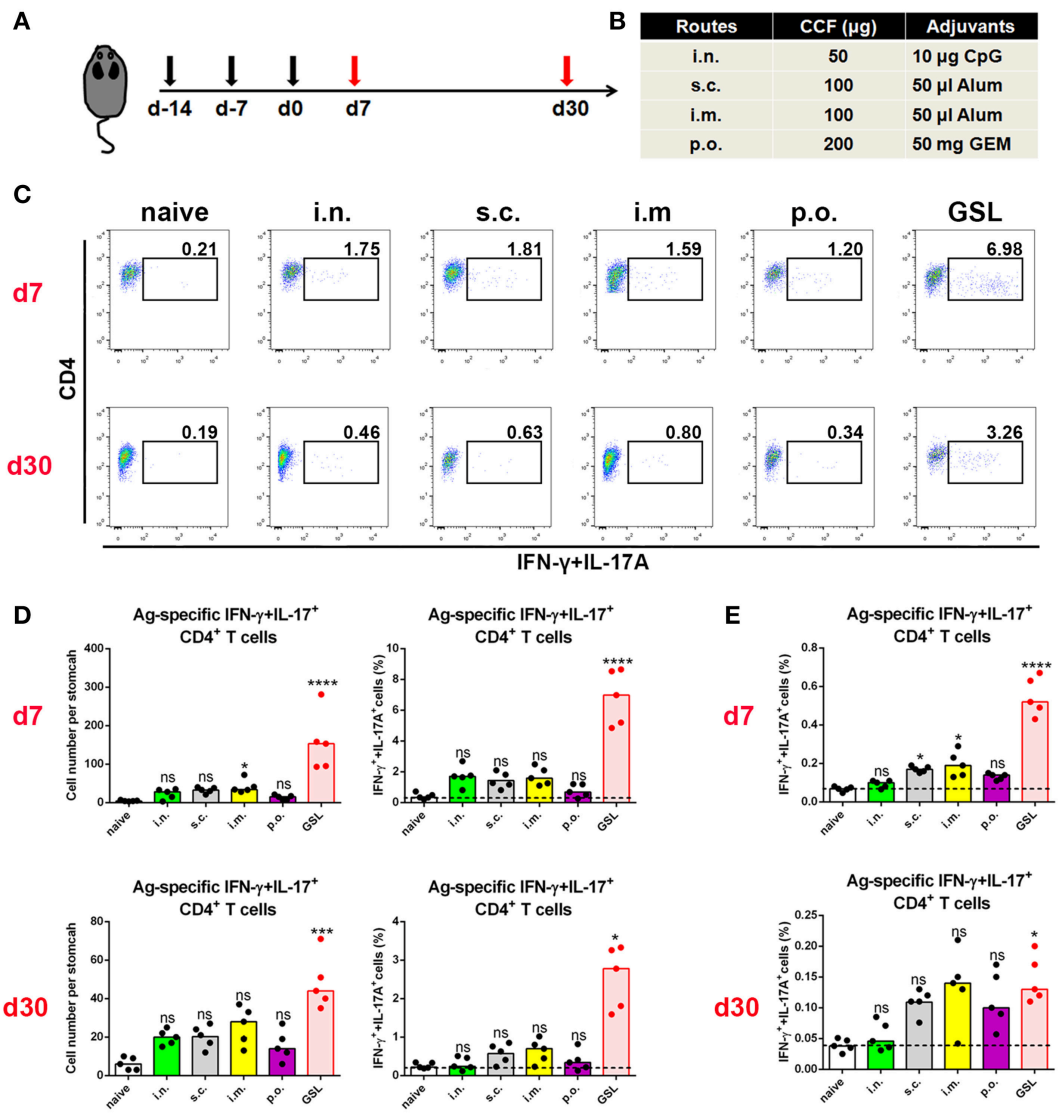
Conventional vaccinations failed to induce a durable Ag-specific CD4+ memory T cell pool in the stomach. **(A)** C57BL/6J mice were immunized at Day-14, Day-7, and Day 0 with different vaccine strategies **(B)**. At Day 7 and Day 30 after the last vaccination, mice were sacrificed and the Ag-specific CD4+ T cells in stomach were measured by intracellular cytokine staining. Purified cells were restimulated with Ag-preloaded, CFSE-labeled, naïve splenocytes for 12 h in the presence of 5 μg/ml BFA. IFN-γ- and/or IL-17A-producing CD90.2+CD4+ cells were identified as Ag-specific CD4+ T cells. At Day 7 (top) and Day 30 (bottom) after the last vaccination, gastric Ag-specific CD4+ T cells from these immunized mice were analyzed **(C)**. Absolute number and frequencies of gastric Ag-specific CD4+ T cells among total CD4+ T cells at Day 7 (top) and Day 30 (bottom) were quantified **(D)**. The frequencies of Ag-specific CD4+ T cells from MLN among total CD4+ T cells at Day 7 (top) and Day 30 (bottom) were quantified **(E)**. In all graphs, dots represent individual data points and columns represent median values. *P < 0.05, ***P < 0.001, ****P < 0.0001, *ns* = not significant. The Kruskal–Wallis test (vs. naïve) was used. Data were pooled from two individual experiments with *n* = 5 mice per group.

Previous studies suggest that specific vaccinations can evoke a transient state that which allows Teff cell migration into non-lymphoid tissues at effector stage (6, 23). To detect gastric Ag-specific CD4+ T cells after conventional vaccinations, we performed different vaccine administrations on the mice and compared the gastric Ag-specific CD4+ T cells at Day 7 and Day 30 (Figures 1A,B). Naïve mice were used as a negative control to exclude non-specific staining, and mice receiving gastric subserosa layer (GSL) vaccination were used as a positive control. Ag-specific CD4+ T cells could be observed on Day 7 in stomach of mice receiving subcutaneous (s.c.), intranasal (i.n.), intramuscular (i.m.) and oral (p.o.) vaccinations, but the number was much lower than that in the GSL control mice (Figures 1C,D). To investigate whether these Ag-specific CD4+ T cells could form a gastric memory T cell pool, we compared the number of gastric Ag-specific CD4+ T cells among different groups on Day 30. Results revealed that few Ag-specific CD4+ T cells were detected in these groups except for GSL (Figures 1C,D). Moreover, the expansion of Ag-specific CD4+ T cells was observed on Day 7 in the MLN from mice receiving i.m. or s.c. administration (Figure 1E). These findings suggest that conventional vaccine strategies can drive some of Ag-specific CD4+ T cells presenting in stomach, but these cells fail to give rise to a formidable memory T cell pool.

### Gastric Subserosa Layer Vaccination Recruits Abundant Ag-specific CD4+ T Cells Into Stomach

**Figure 2:**
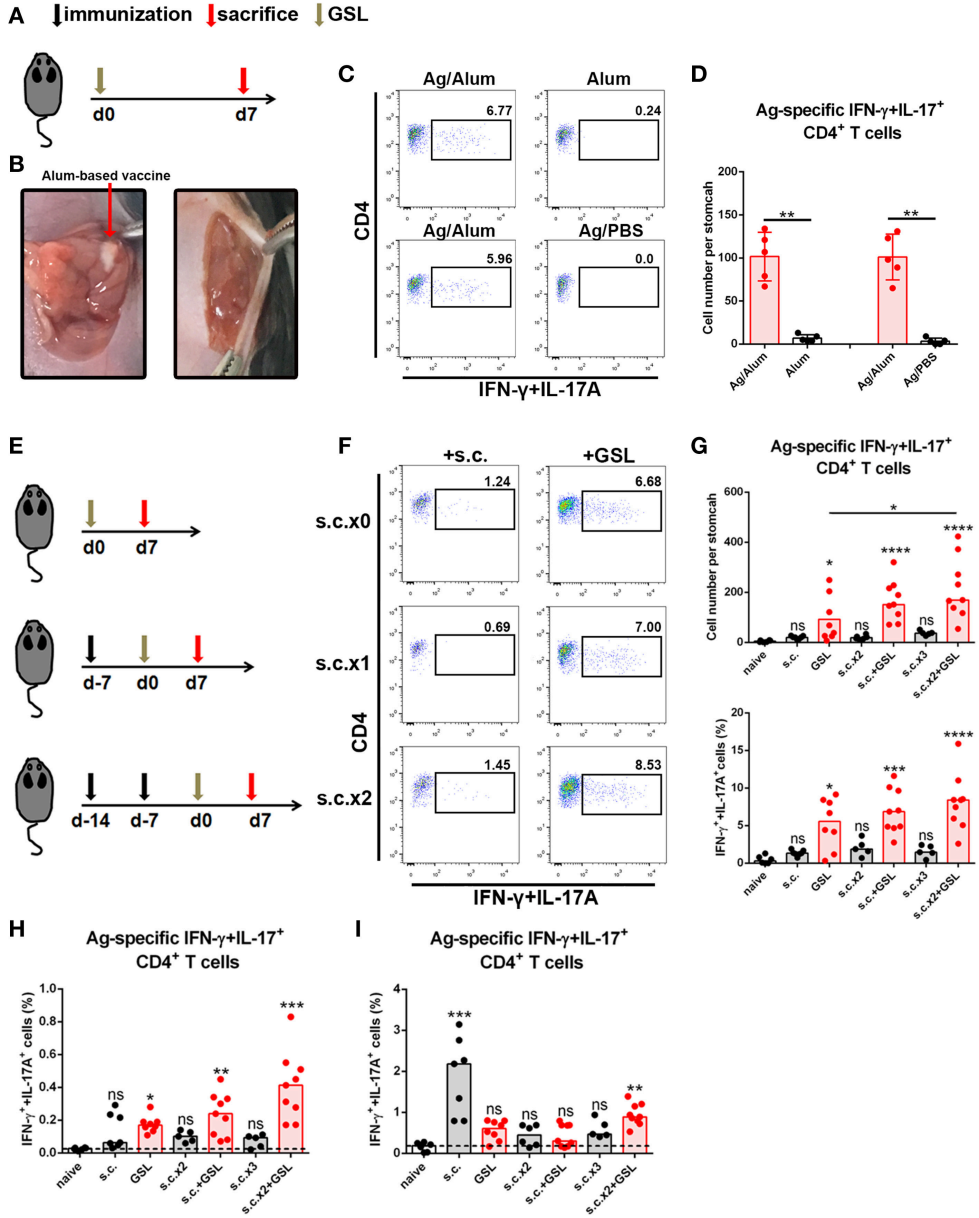
A vaccine strategy triggered abundant Ag-specific CD4+ T cells infiltration of the stomach. **(A)** C57BL/6J mice were immunized at Day 0 by GSL and sacrificed on Day 7. **(B)** Details for GSL vaccination are as follows: an incision was made above the stomach and 5 μl vaccine formulation (containing ~7.5 μg Ag) was injected into the subserosa layer of the stomach. The incisions in peritoneum and skin were sutured. **(C)** Gastric Ag-specific CD4+ T cells were analyzed in mice that GSL vaccinated with Ag/Alum, Alum and Ag/PBS. **(D)** Absolute number of gastric Ag-specific CD4+ T cells at Day 7 were quantified **(E)** C57BL/6J mice were immunized with one of six different strategies. **(F)** Gastric Ag-specific CD4+ T cells in each group were analyzed as described before. Absolute number and frequencies of gastric Ag-specific CD4+ T cells among total CD4+ T cells at Day 7 were quantified **(G)**. The frequencies of Ag-specific CD4+ T cells from MLN **(H)** and spleen **(I)** among total CD4+ T cells were quantified. In all graphs, dots represent individual data points and columns represent median values. *P < 0.05, **P < 0.01, ***P < 0.001, ****P < 0.0001, *ns* = not significant. The Kruskal–Wallis test (vs. naïve) or Mann–Whitney U test (for two groups) was used. Data were pooled from six individual experiments with *n* = 5–9 mice per group.

Development of an *in situ* vaccine strategy might be of utmost importance to establish a strong CD4+ Trm pool in stomach (24, 25). GSL injection has been used for local anesthesia and for the development of gastric ulcer or *in situ* tumor animal models. Given that orientation of visible blood vessels is from lesser curvature to greater curvature, we hypothesized that the non-vascular zone of greater curvature was a feasible region to establish a local Ag depot. To verify this, we formulated CCF with Alum adjuvant, performed the laparotomy to access stomach, and injected 5 μl vaccine into GSL (Figures 2A,B). Acute inflammation, which was characterized by mucosal swelling, was observed in the vaccination site, and abundant Ag-specific CD4+ T cells could be detected 5–7 days later. To clarify the requirement for Ag-specific CD4+ cell recruitment in GSL vaccination, mice were injected with Ag/Alum, Alum alone, or Ag/PBS solution, respectively. Injection of Alum alone failed to recruit any Ag-specific CD4+ T cells, but induced regional tissue swelling (Figures 2C,D). In comparison, GSL injection of Ag/PBS solution did not recruit Ag-specific CD4+ T cells into stomach either, and no tissue alteration was observed in the injection site. We found that Ag/PBS solution could be absorbed within hours after injection, indicating that a sustained-release vehicle (i.e., Alum adjuvant) was necessary for Ag-specific T cell recruitment.

In consideration of the low dosage of vaccine used in GSL vaccination, we modified the vaccine strategy with an additional step to create the following protocol: i) subcutaneous vaccination to induce a systemic Ag response, followed by ii) a surgical operation to inject a micro-dose of vaccine into GSL. To test this strategy for the induction of an Ag-specific T cell response, we performed six kinds of vaccination programs that included multiple rounds of subcutaneous vaccination and/or a GSL vaccination (Figure 2E). As expected, some of gastric Ag-specific CD4+ T cells were detected in mice receiving subcutaneous vaccination alone, but abundant these cells were observed in GSL-vaccinated mice. Performing two rounds of subcutaneous vaccination significantly boosted the number of Ag-specific CD4+ T cells recruited by GSL vaccination (Figures 2F,G). We also examined the percentages of Ag-specific CD4+ T cells in spleen and MLN. s.c. vaccination did not induce plenty of Ag-specific CD4+ T cells in MLN, whereas Ag-specific CD4+ T cells were abundant in mice receiving s.c. vaccination plus GSL vaccination (Figure 2H). In the different vaccination programs, the levels of splenic Ag-specific CD4+ T cells were variable (Figure 2I). We also tested whether GSL injection of mice with Alum or Ag/PBS solution after s.c. vaccination could induce plenty of Ag-specific CD4+ T cells into stomach. The results showed that without regional Ag exposure and adjuvant-induced inflammation, recruitment of Ag-specific CD4+ T cells was limited (Supplementary Figure 2). These results indicated that introduction of a systemic Ag-specific response enhanced the regional Ag-specific CD4+ T cell recruitment induced by GSL vaccination.

Taken together, these data indicate that the local Ag encounter, Ag vehicle phase and systemic immune response all contribute to the maximum recruitment of Ag-specific CD4+ T cells induced by GSL vaccination.

### Ag-specific CD4+ T Cells Retain in Stomach Long-Term Without Recirculation

**Figure 3:**
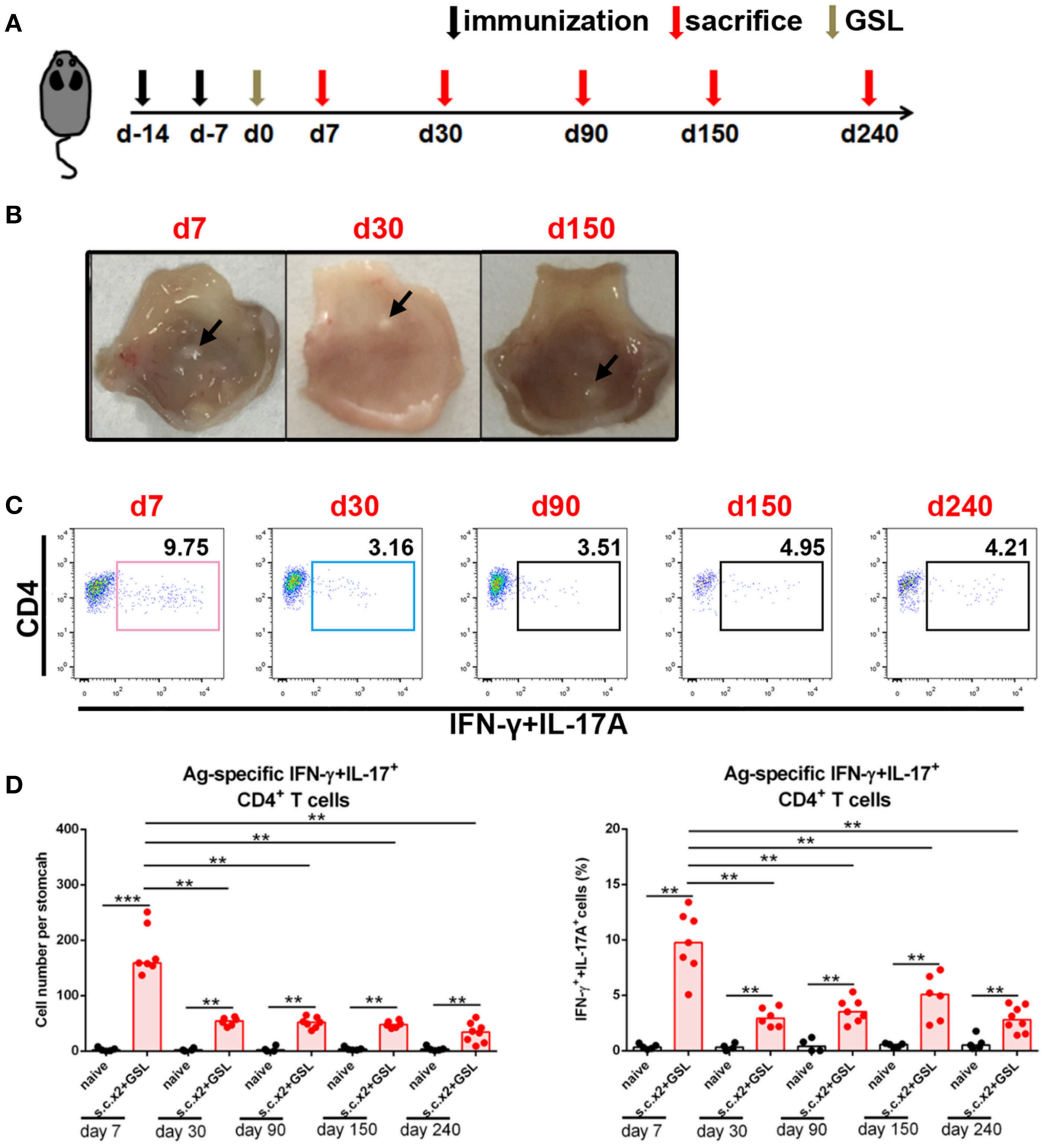
Ag-specific CD4+ Teff cells contracted and sustained in stomach long-term. **(A)** C57BL/6J mice were s.c. immunized with Ag/Alum at Days-14 and-7, and treated with GSL vaccination at Day 0. **(B)** Mice were sacrificed on Day 7, Day 30, and Day 150, and their stomachs were unfolded. Arrows indicated granulation tissue in the vaccination sites. **(C)** Mice were sacrificed on the day indicated in the timetable (red arrows), and the gastric Ag-specific CD4+ T cells in each time point were analyzed as described previously. **(D)** Absolute number and frequencies of gastric Ag-specific CD4+ T cells among CD4+ T cells at each time points were quantified. In all graphs, dots represent individual data points and columns represent median values. **P < 0.01, ***P < 0.001. Mann–Whitney U test was used to compare two groups. Data were pooled from six individual experiments with *n* = 4–8 mice per group.

To investigate whether a durable Ag-specific CD4+ Trm pool was formed after s.c.x2 +GSL vaccination, we counted the number of Ag-specific CD4+ T cells in stomach over the following 8 months (Figure 3A). Age-matched naïve mice were served as negative controls to exclude the influence of age. In immune mice, the granulation tissue induced by the Alum adjuvant was stiffened at Day 30 and shrank in the following months (Figure 3B). No gland atrophy or metaplasia was observed in the mucosa near the vaccination site during Days 30–60 (Supplementary Figure 3). On the other hand, contraction of the infiltrating Ag-specific CD4+ T cell population was complete within 30 days and a stable Ag-specific CD4+ memory T population could be detected for at least 240 days (Figures 3C,D).

**Figure 4:**
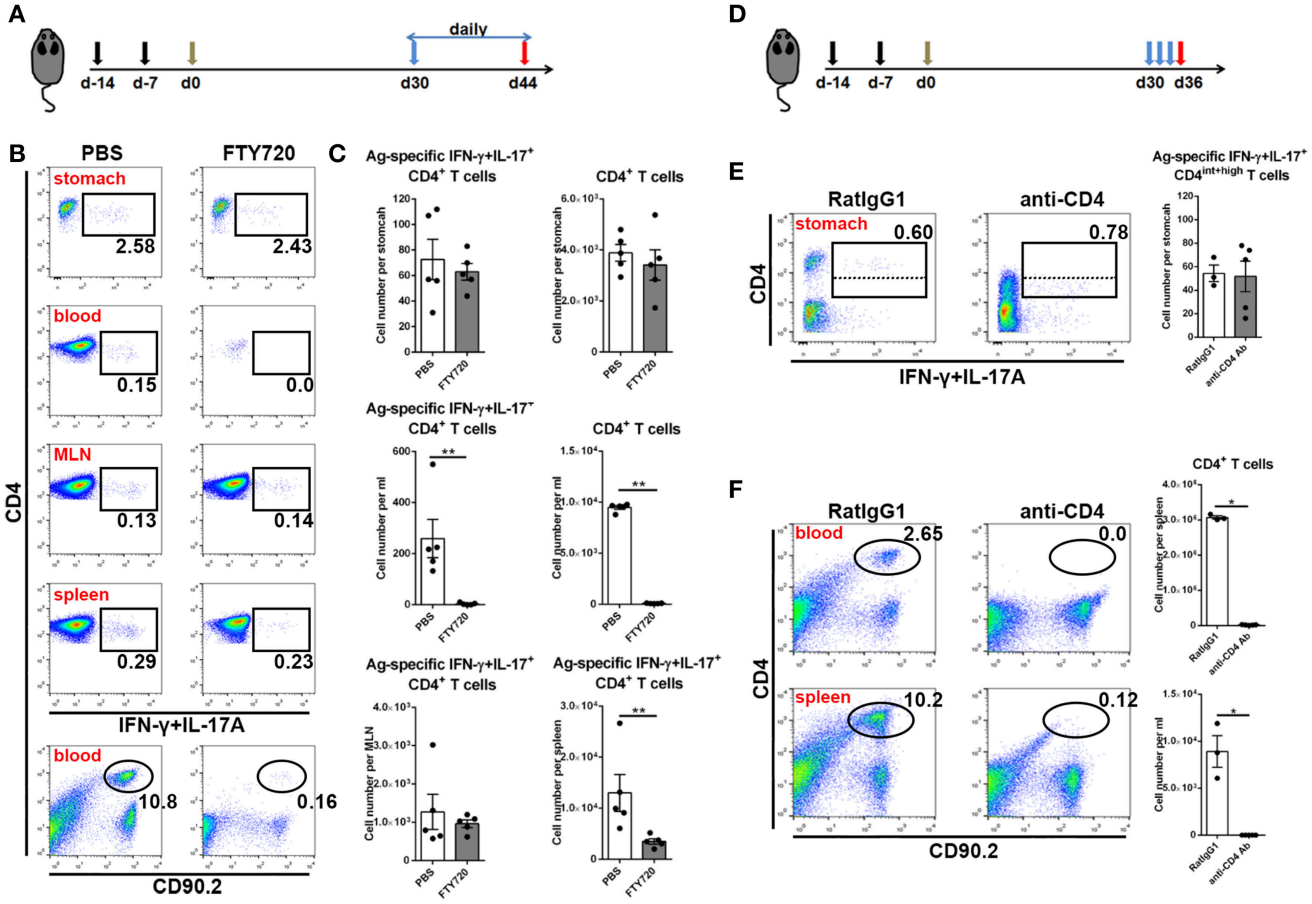
Gastric Ag-specific CD4+ memory T cells induced by GSL vaccination experienced little recirculation and were located separately from the blood. **(A)** C57BL/6J mice were s.c. immunized with Ag/Alum at Days-14 and-7, and treated with GSL vaccination at Day 0. Thirty days later, the mice were i.p. injected with 1 mg/ml of FTY720 or PBS daily for 14 days. **(B)** Ag-specific CD4+ T cells among CD4+ T cells in the stomach, blood, MLN and spleen and CD4+ T cells among total leukocytes were analyzed. **(C)** Absolute number of Ag-specific CD4+ T cells in stomach, MLN, blood and spleen and CD4+ T cells in stomach and blood at the whole organ level were quantified. **(D)** 30-day post GSL vaccination, mice were i.p. injected with 100 μg anti-CD4 antibody or anti-RatIgG1 antibody at Days 30, 32 and 34. Mice were sacrificed on Day 36. **(E)** Ag-specific CD4+ T cells among total CD90.2+ cells in stomach were shown (left), and the absolute number of Ag-specific CD4+ T cells in stomach was quantified (right). **(F)** CD4+ T cells among total leukocytes in blood or spleen were shown (left), and the absolute number of CD4+ T cells in blood or spleen was quantified (right). In all graphs, dots represent individual data points and columns represent mean and SEM. *P < 0.05, **P < 0.01. Mann–Whitney U test was used to compare two groups. Data were pooled from two individual experiments with *n* = 3–5 mice per group.

In the next step, to investigate whether these CD4+ memory T cells experienced recirculation via the lymphovascular system, we treated immune mice with FTY720 daily to inhibit lymphocyte egress from lymph nodes for 14 days (Figure 4A). Even though circulating CD4+ T cells were decreased by more than 100-fold in blood and Ag-specific CD4+ memory T cells vanished from blood, the number of Ag-specific CD4+ memory T cells was stable in stomach (Figures 4B,C), suggesting a characteristic of local retention. Moreover, the absolute number of Ag-specific CD4+ memory T cells was not altered in MLN, but significantly decreased in spleen after FTY720 administration (Figures 4B,C).

To verify whether these Ag-specific CD4+ Trm cells were sensitive to systemic CD4 antibody depletion, we i.p. injected immune mice with anti-CD4 antibody (Figure 4D). No CD4+ T cells could be detected in blood or spleen, whereas Ag-specific CD4+ cells in stomach remained numerically unchanged and measurable, although with lower CD4 expression (Figures 4E,F), implying their retention in a distinct anatomical location which was less affected by circulation. Collectively, these data reveal that infiltrating Ag-specific CD4+ Teff cells can form a long-lived Trm pool and most of these cells may be separated from circulation.

### Distribution of These CD69+CD103-CD4+ Trm Cells Is Dependent on Epithelial Architecture of Stomach

**Figure 5:**
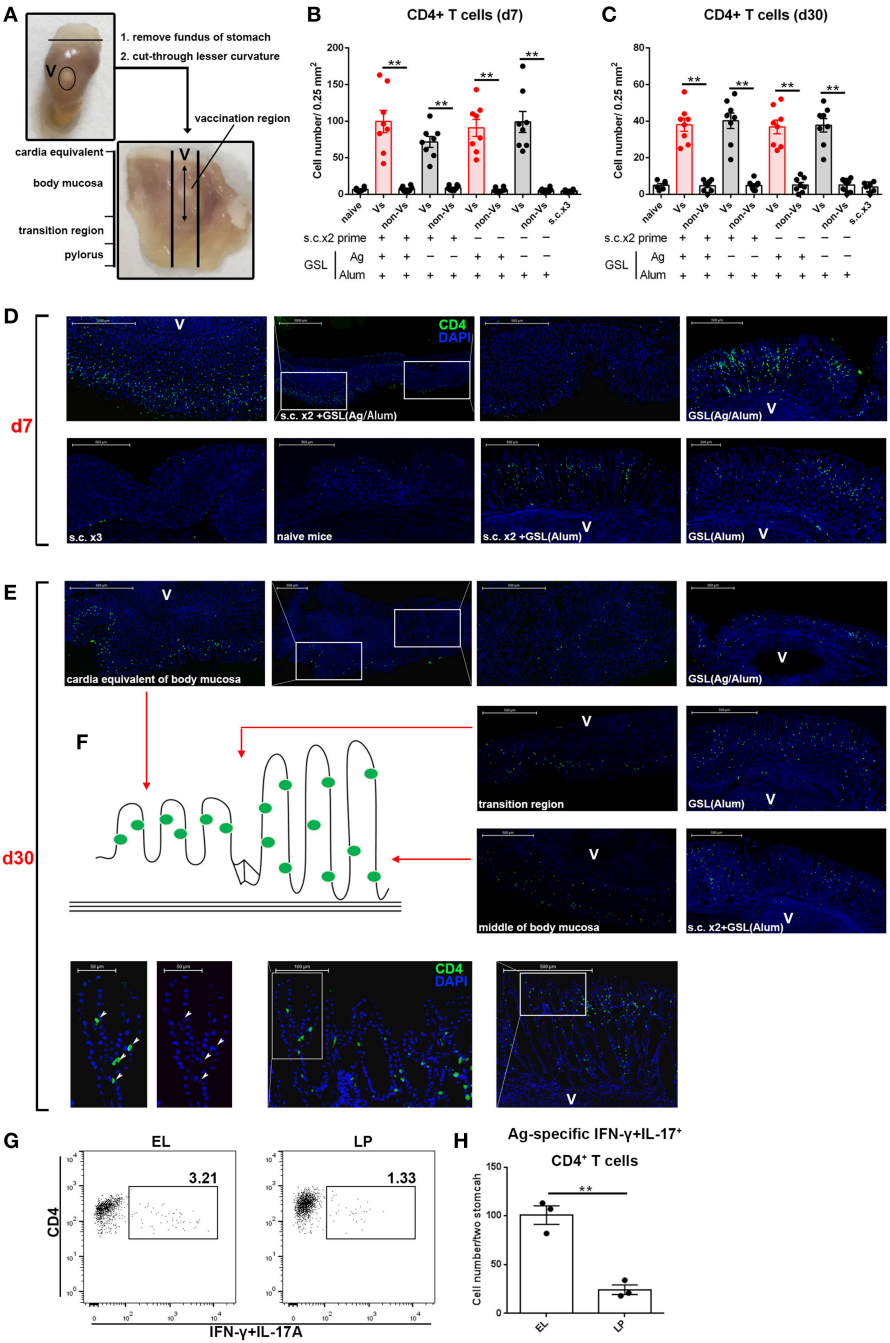
GSL vaccination drove CD4+ T cell infiltration of the mucosa and retention within the epithelium. **(A)** Stomach was dissected as photo indicated and anatomical position was shown in the macroscopic perspective. Gastric tissue from mice that were treated with GSL vaccination was cut longitudinally and stained with Alexa Fluor® 488-anti-CD4 antibody. CD4+ T cells were quantified on immunofluorescent staining of 20 μm frozen sections from Day 7 **(B)** or Day 30 **(C)** post GSL vaccination. **(D,E)** Representative images of immunofluorescent staining (green, CD4; blue, DAPI; V, vaccination site). **(F)** Schematic plan of CD4+ cell location (left) and the distribution of CD4+ cell on gastric middle mucosa and transition region (right). The precise positions of CD4+ cells were determined by immunofluorescent staining of 10 μm frozen sections (green, CD4; blue, DAPI; V, vaccination site). **P < 0.01. Mann–Whitney U test was used to compare two groups. Data were repeated 3–8 times. **(G)** EL or lamina propria (LP) lymphocytes were pooled from two immune mice at memory stage. Ag-specific CD4+ T cells were quantified **(H)**. Six mice (*n* = 6) were used in this experiment. **P < 0.01, unpaired *t*-test.

We next examined the location of CD4+ T cells induced by GSL vaccination. Stomach was isolated and cut through lesser curvature. As shown in Figure 5A, vaccination region was sniped longitudinally and used for immunohistological staining of CD4+ T cells. Six groups with different vaccination programs were involved in this experiment. Mice were sacrificed on 7-day or 30-day post GSL vaccination. The results indicated that abundant CD4+ T cells could be observed in the gastric mucosa near the vaccination site at 7-day post GSL vaccination (Figures 5B,D). Infiltration of CD4+ T cells was restricted to the adjacent mucosa, as few CD4+ cells presented in the mucosa of non-vaccination site. Density of CD4+ T cells was not associated with Ag exposure and prime. Extremely low density of CD4+ T cells was observed in stomach of naïve and s.c. immunized mice in the same perspective. Additionally, only some CD4+ T cells appeared around the vaccination site, even though the surrounding region was enriched with immune cells (Figure 5D). At 30-day post GSL vaccination, density of CD4+ T cells on mucosa of vaccination site was decreased (Figures 5C,E). Notably, at this time point, most CD4+ T cells were located close to the epithelium, which was confirmed by the distinct epithelial architecture of stomach (Figures 5E,F). For instance, in the body mucosa close to the cardia equivalent, CD4+ Trm cells formed a chain; in the middle of the body mucosa, CD4+ Trm cells were evenly distributed in the epithelial region of gastric pit; and in the transition region, both two retention patterns could be observed. Moreover, 10 μm sections of gastric tissue indicated that these CD4+ T cells were in contacted with the epithelial cells (Figure 5E, bottom). Parallelly, detection of Ag-specific CD4+ T cells in the epithelium or lamina propria also suggested that at memory stage, most of Ag-specific CD4+ cells were located on epithelial regions (Figures 5G,H). These data demonstrate that CD4+ T cells recruited by GSL vaccination preferentially infiltrate the adjacent mucosa and survive in the special niches of the epithelium.

**Figure 6:**
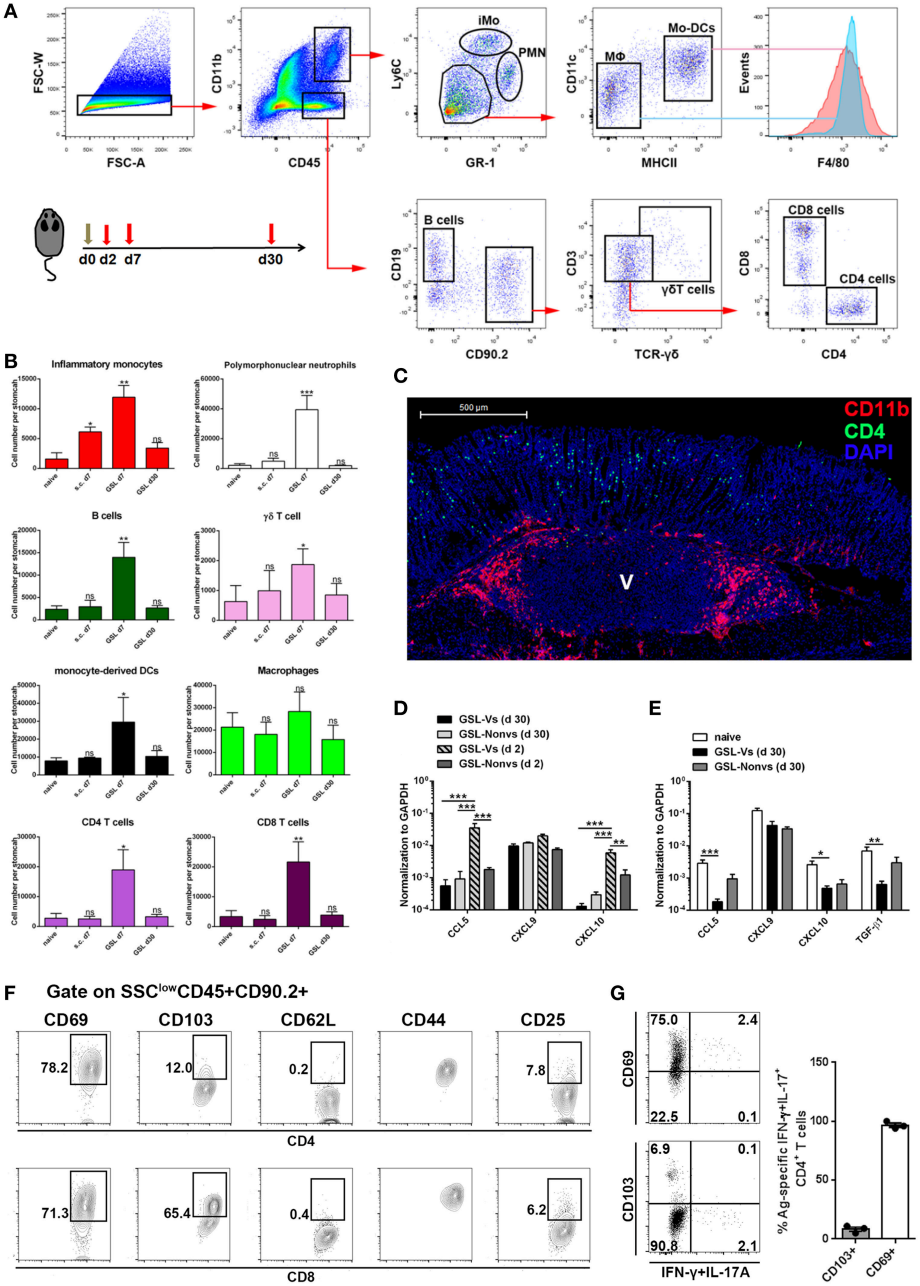
The maintenance and phenotype of CD4+ Trm cells. **(A)** GSL vaccination was performed at Day 0 and mice were sacrificed on Day 7 and Day 30 to analyze immune cell infiltration using the indicated gated strategy. **(B)** Absolute number of innate and adaptive immune cells at the whole organ level was quantified. *P < 0.05, **P < 0.01. The Kruskal—Wallis test (vs. naïve) was used. Columns represent mean and SD. Data were pooled from two individual experiments with *n* = 5–6 mice per group. **(C)** Immunofluorescent staining of 20 μm frozen sections at Day 30 (green, CD4/CD11b; red, CD11b; blue, DAPI; V, vaccination site) post GSL vaccination. Data were repeated at least three times with similar results. **(D)** CCL5, CXCL9, and CXCL10 mRNA were measured at Day 2 and Day 30 by qRT-PCR. GSL-Vs: vaccination site; GSL-Non vs: non-vaccination site. **(E)** CCL5, CXCL9, CXCL10 and TGF-β1 mRNA were measured at Day 30 by qRT-PCR. **(F)** Phenotypes of intraepithelial CD4+ T cells and CD8+ T cells in the vaccination region. **(G)** Expression of CD69 and CD103 was analyzed on Ag-specific CD4+ Trm cells. *P < 0.05, **P < 0.01, ***P < 0.001. Mann–Whitney U test was used to compare two groups. Data were pooled from two individual experiments with *n* = 3–5 mice per group. Columns represent mean and SEM.

Previous studies suggest that CD4+ Trm cells are maintained in vagina, intestine and skin, and cluster with macrophages, dendritic cells (DCs) and CD8+ T cells (24, 26, 27). To examine whether other immune cells were responsible for CD4+ Trm cell residence in stomach, we analyzed the types of infiltrated immune cells at whole organ level on Day 7 and Day 30 (Figure 6A). All cell types except macrophages, i.e., neutrophils, inflammatory monocytes, mo-DCs, B cells, γδ T cells, CD4+ T cells and CD8+ T cells, were expanded on Day 7 (Figure 6B). However, flow cytometric analysis showed that most of the immune cells seceded from stomach before Day 30, suggesting that the increased gastric immune cell content induced by GSL vaccination was not sustainable at the whole organ level. To visualize the regional relationship between CD4+ T cells and innate immune cells, an immunolocalization assay was performed and the images indicated no direct relationship between the CD4+ and CD11b+ cells during the memory stage, as the CD11b+ cells preferentially enveloped the vaccination site (Figure 6C). Next, we measured several chemokines CCL5, CXCL9 and CXCL10 that are critical for CD4+ T cell recruitment (26). Compared with memory stage, CCL5 and CXCL10 levels were significantly increased in the vaccination site during effector stage (Figure 6D). Interestingly, at memory stage, CCL5, CXCL10, and TGF-β1 levels at the vaccination site were lower than those of naïve mice (Figure 6E). We reckoned that the granuloma structure might affect regional homeostasis. In total, these data demonstrate that a distinct migration and retention pattern of CD4+ T cells is induced by GSL vaccination.

Next, we isolated intraepithelial T cells from the vaccination site and analyzed their phenotypes at the memory stage. CD4+ T cells from the vaccination site expressed CD69 and CD44, but expressed little CD103 and no CD62L; on the contrary, CD8+ T cells in this region expressed CD103, CD69, and CD44 (Figure 6F). Parallelly, we measured the expression of CD69 and CD103 on Ag-specific CD4+ Trm cells. Almost all these cells displayed a CD69+CD103-phenotype (Figure 6G). These data indicate that GSL vaccination induces intraepithelial CD69+CD103- CD4+ Trm cells in stomach.

### Vaccine Strategy Involving CD4+ Trm Cells Provides Rapid and Long-Term Protection Against *Helicobacter* Insult

**Figure 7:**
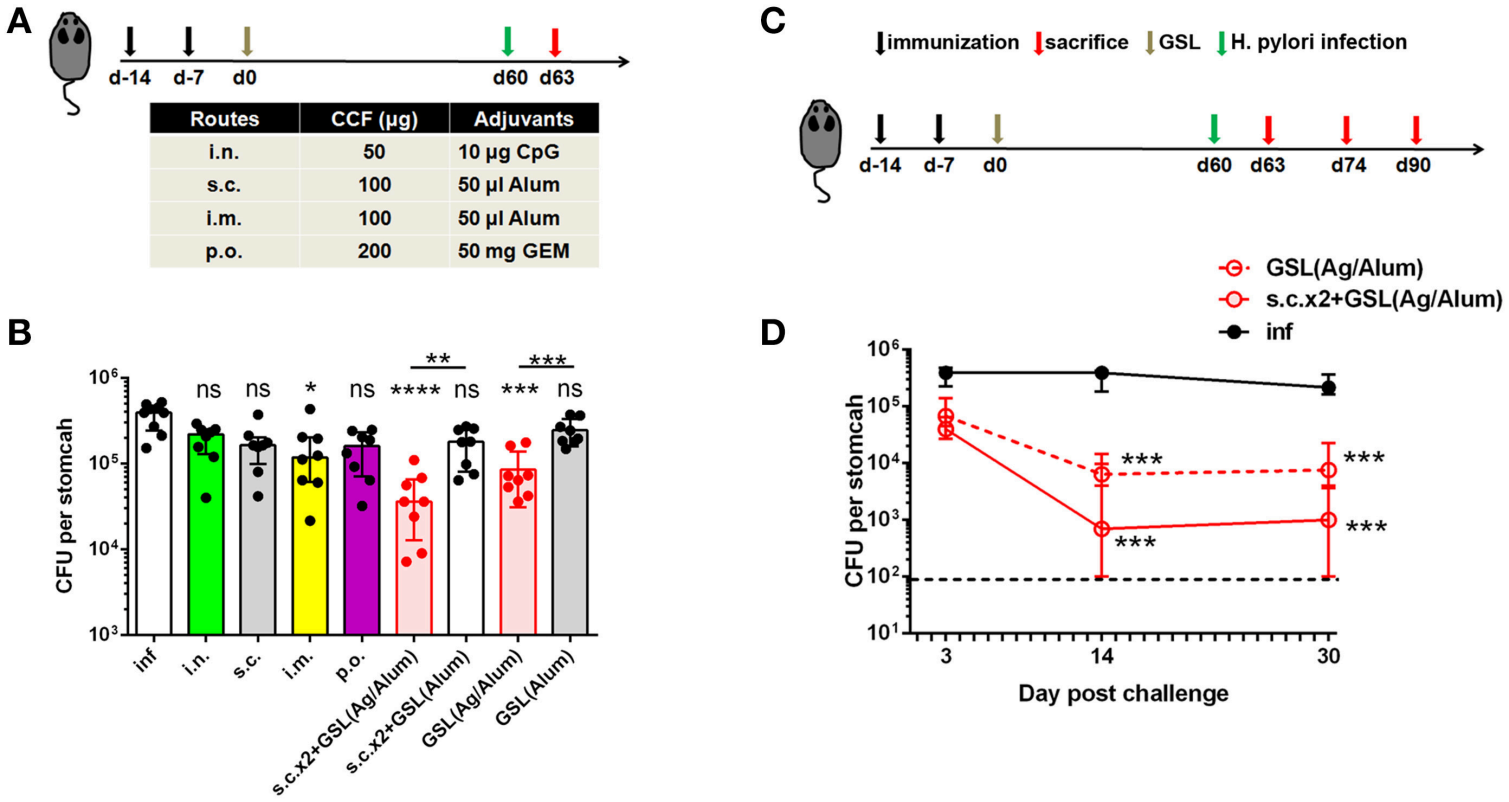
s.cx2+GSL vaccination provided prolonged and rapid protection against *Helicobacter* insult. **(A)** C57BL/6J mice were immunized at Day-14, Day-7, and Day 0 with different vaccine formulation described in the table. *Helicobacter* challenge was performed on Day 60 and mice were sacrificed on Day 63 to test the protective effects. **(B)** *H. pylori* colonization was determined by the quantitative culture on Day 63. *P < 0.05, ***P < 0.001, ****P < 0.0001, *ns, P* > 0.05. The Kruskal–Wallis test (vs. naïve) was used. Data were pooled from two individual experiments with *n* = 8 mice per group. **(C)** Mice experienced s.cx2+GSL vaccination were challenged at Day 60 and H. pylori colonization was determined on Day 63, Day 74, and Day 90 **(D)**. **P < 0.05, ***P < 0.01. Mann–Whitney U test was used to compare two groups. In all graphs, columns represent median and interquartile. Data were pooled from two individual experiments with *n* = 6–8 mice per group.

Vaccination programs used in previous studies generally performed *H. pylori* challenge on ~14 days after the last vaccination (28, 29). At this time point, Teff cells induced by conventional vaccinations might not completely attenuate in blood or stomach of immune mice. A previous clinical study indicated that the protective effects induced by oral vaccination continued to attenuate in the years that followed (30). Here, we tested the differences between conventional vaccinations and vaccine strategies involving GSL vaccination in terms of rapid and long-term protection. Mice were divided into nine groups and subjected to different immunization programs (Figure 7A). *H. pylori* challenge was performed 60 days later. At Day 63, colonization of *H. pylori* was determined by quantitative culture. Substantial reductions of bacterial colonization could be found in mice that received GSL vaccination, and s.c.x2+GSL vaccination provided the optimal protection at this time point (Figure 7B), indicating that these mice demonstrated rapid antimicrobial responses and prolonged protection. In comparison, most conventional vaccine strategies failed to reduce bacterial colonization and no reduction of *H. pylori* colonization was observed in mice receiving Alum alone by GSL vaccination. Furthermore, we investigated the limit of anti-microbial response involving CD4+ Trm cells (Figure 7C). Results showed that s.c.x2+GSL vaccination induced a drastic reduction of *H. pylori* load at day 0–14 and the bacterial load was sustained at a low degree during day 14–30 post challenge (Figure 7D). A numerical advantage of reduction was observed between s.c.x2+GSL vaccination and GSL vaccination. These results imply that s.c.x2+GSL vaccination induces rapid and long-term protection against *H. pylori*.

### Reactivated CD4+ Trm Cells Trigger a Rapid Systemic Th1/Th17 Cellular Response to Support Tissue-Wide Anti-microbial Response

**Figure 8:**
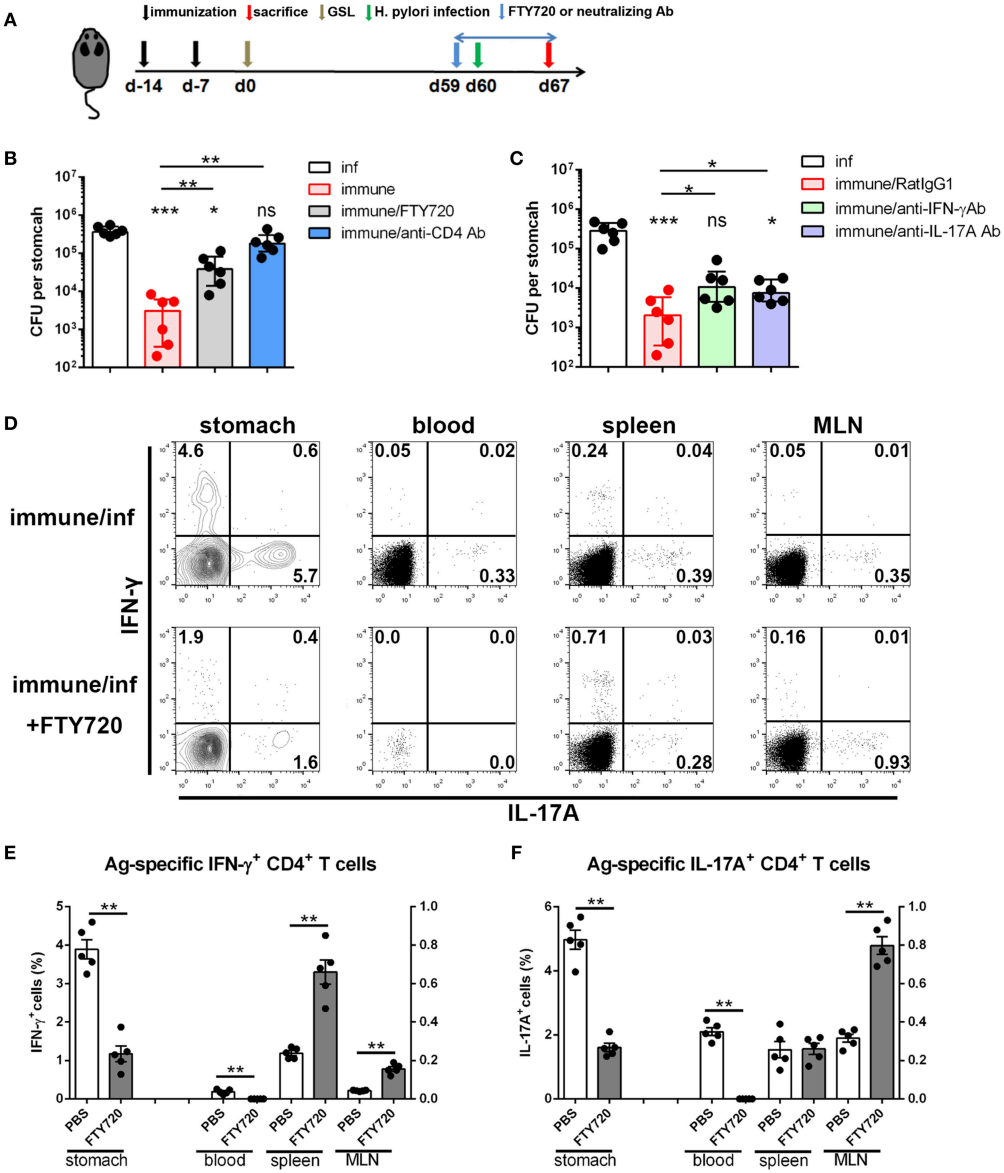
The immunological mechanism of protective response in the mice experienced s.c.x2+GSL vaccination. **(A)** Mice experienced s.cx2+GSL vaccination. *H. pylori* challenge was performed on Day 60. FTY720 administration (1 mg/kg) was performed daily during Days 59–67. Neutralizing antibody (i.p.) injection was performed on Days 59, 61, 63, and 65. *H. pylori* colonization were determined on Day 67 after FTY720 and anti-CD4 antibody administration **(B)** or after anti-IFN-γ and anti-IL-17A antibodies administration **(C)**. *P < 0.05, ***P < 0.001, *ns* = not significant. The Kruskal–Wallis test (vs. naïve) was used or The Mann–Whitney U test was used to compare two groups. Dots represent individual data points and columns represent median and interquartile. Data were pooled from two individual experiments with *n* = 6 mice per group. **(D)** Immune mice were administrated with 1 mg/kg FTY720 daily during Days 59–67 and sacrificed on Day 67 for Ag-specific CD4+ T cell analysis. The percentages of Ag-specific IFN-γ **(E)** or IL-17A **(F)** CD4+ T cells among total CD4+ T cells in stomach, blood, spleen, and MLN were quantified. **P < 0.01, The Mann–Whitney U test was used to compare two groups. Dots represent individual data points and columns represent mean and SEM. Data were pooled from two individual experiments with *n* = 5 mice per group.

Next, we investigated the immunological mechanism of protective response and the role of circulating lymphocytes in mice receiving s.c.x2+GSL vaccination. Administration of FTY720 to immune mice impaired the protective effects, and depletion of CD4+ T cells by an anti-CD4 antibody completely abrogated protection (Figure 8A), indicating the vital roles of circulating memory lymphocytes and CD4+ cells in protective response induced by GSL vaccination. Furthermore, IFN-γ and IL-17A depletion by neutralizing antibody partly suppressed the protective response in immune mice, highlighting the roles of IFN-γ and IL-17A in providing protection (Figure 8C). Analysis of Ag-specific immune response at day 3 post challenge suggested that a potent Ag-specific Th1/Th17 cell response in stomach and the expansion of Ag-specific Th17 cells in spleen already could be detected (Supplementary Figure 4A). Next, we compared the Ag-specific Th1/Th17 cell responses in immune mice treated with or without FTY720 in stomach, blood, spleen, and MLN at 7-day post challenge. FTY720 administration significantly dampened the expansion of Ag-specific Th1 and Th17 cells in stomach and blood (Figures 8D–F). In addition, after preventing lymphocyte egress from the lymph nodes with FTY720, the percentages of Ag-specific Th1 cells in spleen and MLN were increased by 3-fold; however, an increase in the percentage of Ag-specific Th17 cells was only observed in MLN (Figures 8D–F). Because of the powerful ability of Th17 cells to induce an anti-microbial response, reactivated MLN-settled Ag-specific memory Th17 cells might be a vital source of gastric Th17 cells that contribute to the recruitment of innate inflammatory cells and trigger tissue-wide protection. In fact, at 7-day post challenge, the mucosa near the vaccination site showed fewer inflammatory cell infiltration as compared with the distal mucosa (Supplementary Figure 4B), possibly suggesting a tissue-wide inflammatory cell infiltration was temporally delayed to the regional infiltration. These data highlight the alarming function of these intraepithelial CD4+ Trm cells, and indicate that regionally positioned CD4+ Trm cells can trigger tissue-wide *H. pylori* clearance through the recruitment of circulating Th1/Th17 cells.

## Discussion

Stomach is an inhospitable digestive organ that is inhabited by only ~200 different species (8). The notorious one is *H. pylori*, which is a helical rod-shaped organism that is in contact with the gastric epithelium and influences the physiology of gastric stem cell pool by inducing chronic inflammation (31). Evidence indicates that as long as 50,000 years of co-evolution with human have conferred multiple capabilities (e. g., secretion of virulence factors and remodeling of autologous constituents) on *H. pylori* to adapt to the milieu of stomach and escape the host defensive mechanisms (32–35). Likewise, these capabilities lead to an undesirable outcome for *H. pylori* vaccines. Conventional vaccine strategies have been extensively tested in the field of *H. pylori* vaccinology (15, 16, 36–38), while no study identified an Ag-specific cellular response in stomach. Here, we demonstrated that by using intracellular cytokine staining, a small population of Ag-specific CD4+ T cells could be measured during effector stage rather than memory stage of these immunizations, suggesting conventional vaccine strategies were less effective to induce a measurable CD4+ Trm pool for further investigation. Technological barrier for identifying endogenous Ag-specific CD4+T cells in stomach is obvious. The inefficiency of lymphocyte isolation from stomach of mice has already been proved that 98.3% lymphocytes loss in stomach using flow cytometry for counting (2, 39). A need for purification to *ex vivo* culture and extensive steps of intracellular cytokine staining may lead to the additional cell loss (40). Thus, we decided to establish a mice model with a measurable CD4+ Trm pool.

Past studies have revealed that almost always Trm cells form within the tissue after resolution of inflammation or infection (4, 41). Most *H. pylori* vaccines, including the only licensed *H. pylori* vaccine, are composed of non-infectious *H. pylori* Ags that are poorly immunogenic (30). In consideration of the importance of local inflammation, we employed GSL injection to deliver a micro-dose of Alum-based *H. pylori* vaccine into stomach and subcutaneous vaccination was performed before GSL vaccination to mount the infiltrating Ag-specific CD4+ T cells. This immunization strategy established a stratified immune memory involving the local long-lived CD4+ Trm cells and adjacent lymph node-settled memory T cells. Upon GSL vaccination, the regional dissemination of Ag-specific CD4+ T cells was dependent on local Ag recognition and adjuvant-induced inflammation, as either injection with Ag in PBS or delivering Alum adjuvant alone showed no effect on Ag-specific CD4+ T cell recruitment.

Our results also provided insights of CD4+ Trm cells induced by GSL vaccination in terms of migration properties, location/development, and cell surface phenotype. For migration properties, the magnitude of CD4+ Trm cells was stable after FTY720 administration, suggesting they were accord with the important identification parameter that undergoes little or no recirculation (1). For location/development, we found that in GSL immune mice, CD4+ Trm cells showed a distinct pattern of intraepithelial retention. Three phases of their development can be described as followed: in acute phase, CD4+ T cells infiltrated into the mucosa along with the elevated levels of CCL5 and CXCL10 and expansion of various innate immune cells; during the phase of inflammation resolution, more than 60% of CD4+T cells, including Ag-specific CD4+T cells, withdrew from stomach; in memory stage, CD4+Trm cells were distributed along with the architecture of gastric epithelium and keep stable in magnitude for long-term. An elegant study indicates that vaginal CD4+ Trm cells induced by an attenuated herpes simplex virus 2 sustain in a unique lymphocyte structure, named memory lymphocyte cluster, which is located in parenchyma tissue (26). Half of skin CD4+ T cells persist in peri-follicular clusters that accurately equilibrate with the blood lymphocytes during steady state, and infection can increase the immune cell content of these clusters (27). Our data showed that no lymphocyte clusters were observed in stomach of immune mice in memory stage. Residential pattern of the CD4+ Trm cells in our study was different from the prevailing view that after resolution of infection/inflammation, CD4+ Trm cells are preferentially localized within parenchymal tissues, while CD8+ Trm cells adhere to epithelial layers (1). Current knowledge about Trm cells is primarily obtained from invasive pathogens, which can disseminate into the host organ. Differences on types of vaccine, tissue architecture and inflammatory signaling may be responsible for the outcome of CD4+ Trm cell location/development (1, 25, 42, 43). In addition, the cell surface phenotypes of Ag-specific CD4+ Trm cells isolated from GSL immune mice were exclusively CD69+CD103-. A recent study in the context of *Candida albicans* infection finds that non-recirculating skin CD69+CD4+ Th17 cells are sufficient to trigger sterilizing immunity (44). Also, Ag-specific CD4+ Trm cells reported by N. Iijima and A. Iwasaki's study expressed CD69 but little CD103 (26).

Trm cells within peripheral tissues provide strong protection against pathogenic insult (26, 45, 46). Inducing a potent mucosal immune memory is favored as *H. pylori* restrictedly survive in epithelium of stomach with less invasiveness. During *H. pylori* insult, evenly distributed intraepithelial CD4+ Trm cells are optimally positioned to eliminate the window period and initiate a protective response immediately. GSL vaccination-induced CD4+ Trm cells are long-lived and pathogen-specific, therefore providing prolonged protection that was highly sensitive to *H. pylori* insult. As Th1 and Th17 immunity was highlighted in the anti-microbial response of *H. pylori* vaccine (28, 29), we reported that CD4+ Trm cells induced by GSL vaccination sustained in stomach for long-term and rapidly reactivated to recruit circulating Th1/Th17 cells to clear gastric *H. pylori*. Tissue-autonomous protection was found in the immune mice, but superior antimicrobial effects were dependent on the engagement of circulating lymphocytes (Figure 8B). It might be that protective CD4+ Trm cells induced by GSL vaccination were restricted to adjacent mucosa at low magnitudes and needed the help of circulating Th1/Th17 cells to trigger tissue-wide protection (41). This observation is consistent with previous study that small numbers of Trm cells trigger an antiviral state through amplifying innate or adaptive immune signals (47).

In the present study, we attempted to introduce a vaccine-induced CD4+ Trm pool in stomach and evaluated its protective efficacy. Alum-based vaccine was used for GSL vaccination, but we found an undesirable granuloma that affected regional homeostasis. Employing a biocompatible vehicle with the characteristics to spread in GSL is interested to improve the outcome of this model. Our further study will use the silk fibroin to replace Alum adjuvant to extend the CD4+ Trm cell distribution, increase their magnitudes, and prevent the form of granuloma. A more attractive but challenging question is how to design a feasible delivered system that targets stomach. The laparotomy used in mice is impractical in humans. Combining endoscopy technology with GSL vaccination is also less feasible for the translational application. Recently, an impressive study reports an ingestible self-orienting system for oral delivery of macromolecules that deliveries insulin through gastric mucosa (48). This delivered system is applicable for stomach-targeted vaccination after some adjustment.

Overall, our study developed a mice model with a strategic CD4+ Trm pool in stomach. CD4+ T cells induced by GSL vaccination preferentially infiltrated the adjacent mucosa, and then restrictively sustained in the epithelial region adjacent to the vaccination site. The underlying mechanism of local maintenance is currently unknown but may be associated with the metabolism of free fatty acids and TGF-β signals, similar to the maintenance requirements for intraepithelial CD8+ Trm cells (49). Principally, our results indicate that pathogen-specific CD4+ Trm cells within the gastric epithelium can catch the best chance to sound the alarm, orchestrate the defense response, and provide prolonged protection. The notion that developing vaccine strategies involves a Trm population may shed new light on the development of *H. pylori* vaccines.

## Ethics Statement

All animal experiments were approved by the Animal Ethical and Experimental Committee of China Pharmaceutical University.

## Author Contributions

WL and YX designed all experiments. WL performed surgical operation. ZZ, SL, CH, NX, and AH conducted the animals. WL, ZZ, and SL analyzed the data. ZZ prepared the reagents and the experiments. WL, YX, LZ, ES, and TX discussed the results and wrote the manuscript. All authors have reviewed this manuscript before submission.

### Conflict of Interest Statement

The authors declare that the research was conducted in the absence of any commercial or financial relationships that could be construed as a potential conflict of interest.
