# Supplementary material for: Gastric Subserous Vaccination With Helicobacter pylori Vaccine: An Attempt to Establish Tissue-Resident CD4+ Memory T Cells and Induce Prolonged Protection
Source: Front Immunol. 2019 May 17;10:1115. doi: 10.3389/fimmu.2019.01115 (PMC6533896; doi:10.3389/fimmu.2019.01115)
Supplement: Supplementary file 1 [file Data_Sheet_1.PDF]

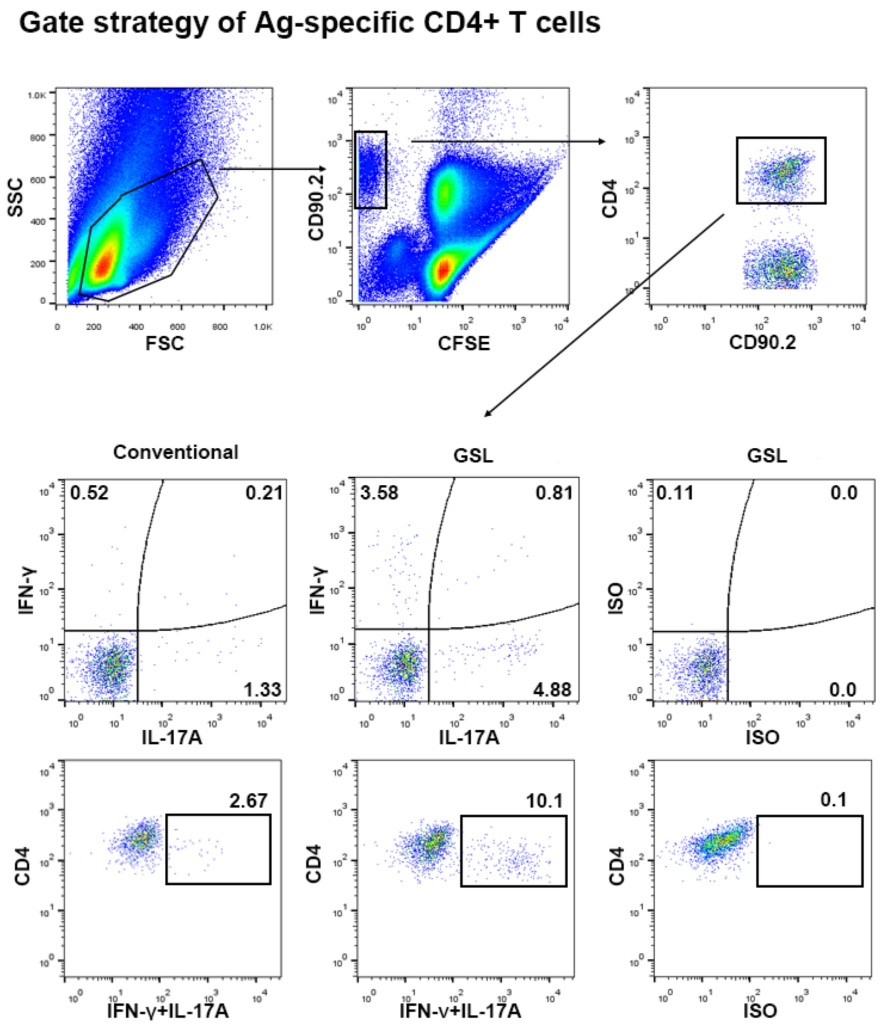


Supplementary Fig. 1 The gate strategy of Ag-specific CD4+ T cells. GSL-positive mice received one dose of GSL vaccination with 5 μl Ag/Alum. Conventional vaccination mice received three rounds of s.c. vaccination with 100 μl Ag/Alum.

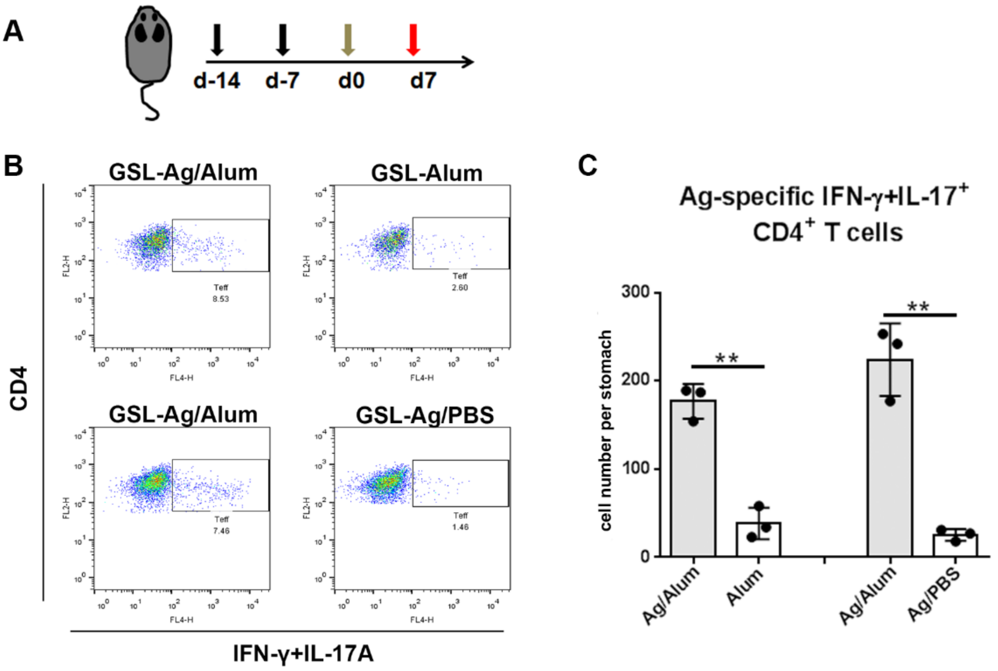


Supplementary Fig. 2. Prime s.c. vaccination did not increased the recruitment of Ag-specific CD4+ T cells after GSL vaccination with Alum or Ag/PBS. (**A**) Mice were s.c. immunized with Ag/Alum two rounds, followed by GSL injection with Ag/Alum, Alum, or Ag/PBS. Mice were sacrificed on Day 7 and the Ag-specific CD4+ T cells in stomach were analyzed (**B**) and quantified (**C**). n=3 mice per group. **P<0.01, unpaired t-test.

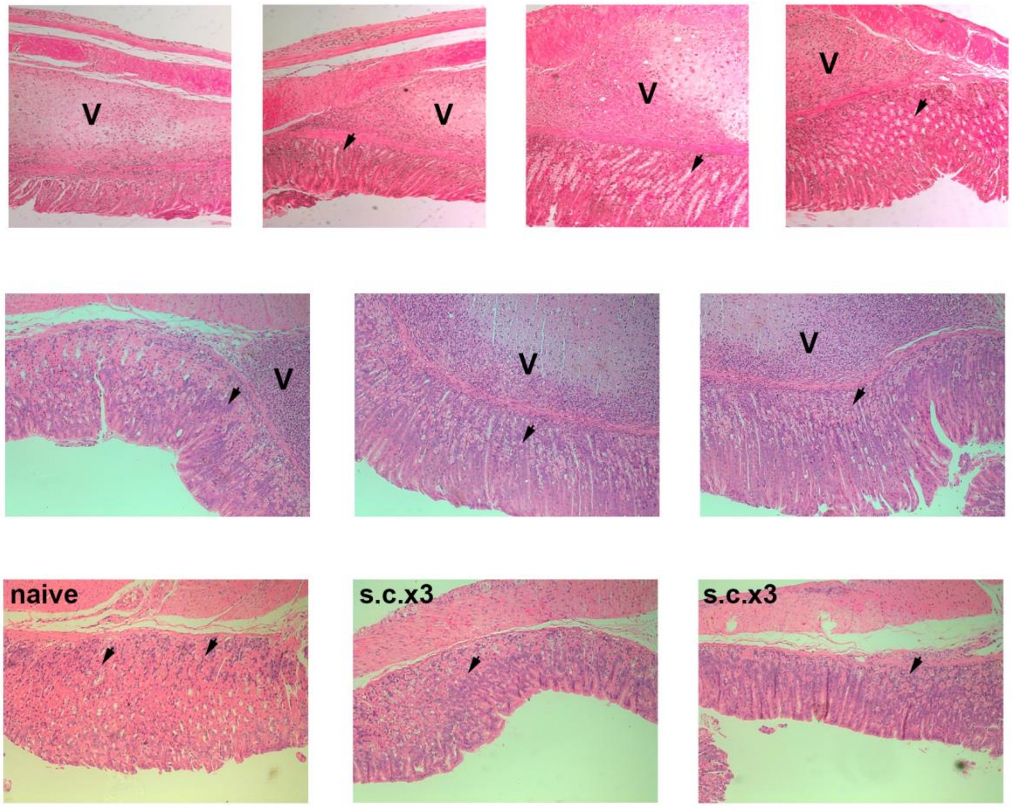


Supplementary Fig. 3 Evaluation of histological alteration in the vaccination site. Mice were sacrificed on Day 30-60 post GSL vaccination and histology was evaluated in stomach. Except for granulation tissue, no pathological alteration of mucosa was found in vaccination site (top and middle). The gastric sections from s.c. immunized mice and naïve mice (bottom). V, vaccination site; Arrows, gastric glands. n=3-5 mice per group.

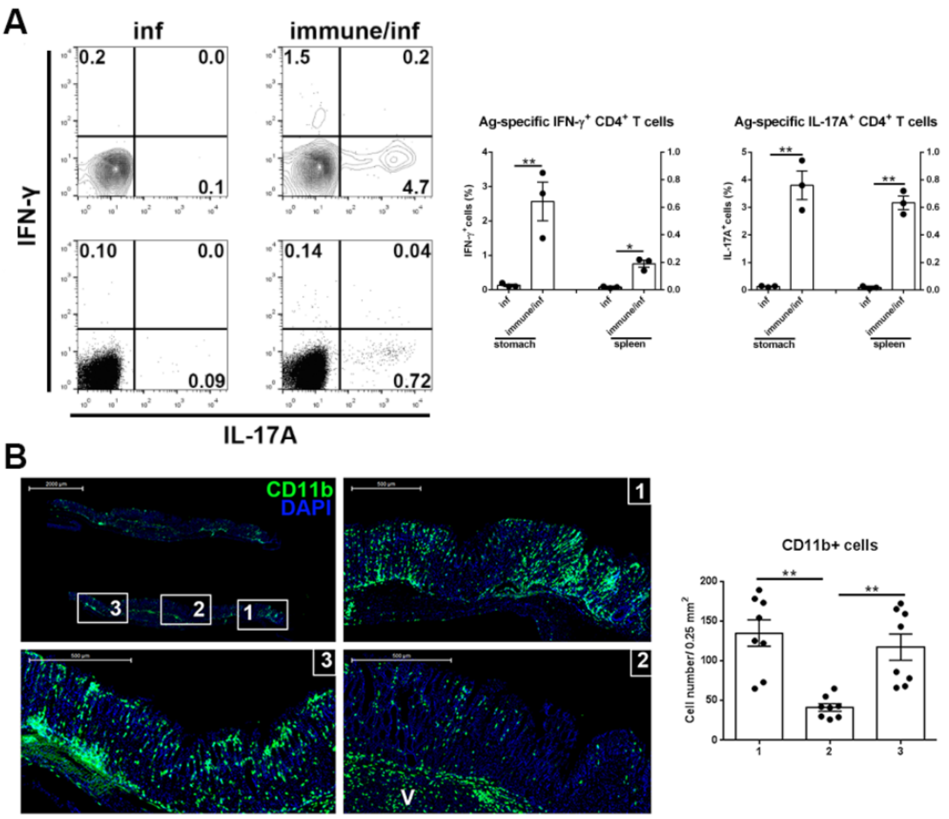


Supplementary Fig. 4 Reactived-Th1/Th17 cells recruited innate inflammatory cells to trigger tissue-wide protection. (**A**) Immune mice were sacrificed on Day 63 and the Ag-specific CD4+ T cells in stomach (upper) and spleen (bottom) were analyzed. The frequency of Ag-specific IFN-γ+ or IL-17A+ CD4+ T cells was analyzed. Data were repeated at 3 mice. **P<0.01, unpaired t-test. (**B**) Mice were sacrificed 7 days post challenge. The sections of stomach were stained with CD11b antibody (green). Abundant CD11b+ immune cells were found in the mucosa and the number of CD11b+ cells in indicated regions (1, distal mucosa; 2, adjacent mucosa; 3, distal mucosa) was quantified. **P<0.01, Mann–Whitney U test was used to compare two groups. Data were pooled from eight sections from three mice.
